# Ocular Safety of Unilateral Biportal Endoscopic Spinal Surgery: An Optical Coherence Tomography Angiography-Based Analysis

**DOI:** 10.3390/jcm15051774

**Published:** 2026-02-26

**Authors:** Ali Gulec, Ebubekir Eravsar, Sadettin Ciftci, Abdullah Beyoglu, Bahattin Kerem Aydin

**Affiliations:** 1 Department of Orthopedics and Traumatology, Selcuk University, Konya 42130, Türkiye; bekireravsar@gmail.com (E.E.); dr.sadettinciftci@gmail.com (S.C.); bkaydin@yahoo.com (B.K.A.); 2 Department of Ophthalmology, Selcuk University, Konya 42130, Türkiye; drabeyoglu@gmail.com

**Keywords:** unilateral biportal endoscopy, UBE, spinal surgery, optical coherence tomography angiography, OCTA, retinal microvasculature, ischemic optic neuropathy

## Abstract

**Background**: UBE has gained popularity as a minimally invasive alternative to open spinal procedures. However, it raises concerns about potential ocular complications. Despite these concerns, there is a lack of studies evaluating UBE’s impact on retinal microvasculature using objective imaging tools such as OCTA. This study aims to evaluate the effects of UBE on the microvascular structures of the retina and optic nerve using OCTA, and to determine whether UBE poses a risk for perioperative vision loss. **Methods**: This study included 32 patients who underwent UBE for lumbar stenosis and received ophthalmologic examinations preoperatively, and at postoperative weeks 1 and 4. Patients with systemic or ocular vascular comorbidities were excluded. OCTA parameters including vascular density (VD), foveal avascular zone (FAZ), retinal nerve fiber layer (RNFL), central macular thickness (CMT), and subfoveal choroidal thickness (SFCT) were evaluated using swept-source OCT. **Results**: No patients experienced clinical vision loss. A statistically significant change was observed over time in FAZ (*p* = 0.043), VDd superior (*p* = 0.018), VDd temporal (*p* = 0.032), and RNFLts (*p* = 0.032). However, only VDd superior showed a statistically significant decrease at postoperative week 4 compared to baseline (*p* = 0.050). All other parameters either returned to baseline or showed no significant change. No clinically relevant visual changes were detected. **Conclusions**: In this study, UBE spinal surgery was not associated with clinically evident visual loss or sustained OCTA-detected microvascular alterations during short-term follow-up. These findings should be interpreted as reflecting the absence of detectable short-term changes rather than definitive evidence of ocular safety.

## 1. Introduction

Unilateral biportal endoscopic spinal surgery (UBE) has emerged as an innovative minimally invasive technique addressing the limitations of conventional open and microscopic approaches for lumbar disc herniation and spinal stenosis [1]. While traditional methods offer established outcomes, they impose constraints on visual field and instrument maneuverability. Percutaneous full-endoscopic techniques, despite advantages like smaller incisions and rapid recovery, present technical challenges particularly in bilateral decompression cases where visual limitations become pronounced [2]. UBE uniquely synthesizes the benefits of open and endoscopic approaches through its biportal fluid-mediated environment, yet this very feature introduces potential risks to ocular perfusion due to controlled hydrostatic pressure [3].

Among spinal surgery complications, perioperative vision loss (POVL) remains one of the most devastating, with ischemic optic neuropathy (ION) accounting for 89% of cases [4]. The prone position, prolonged operative time, and hemodynamic shifts are established risk factors, but UBE’s unique requirement for sustained irrigation pressure may further elevate intraorbital pressure, potentially compromising optic nerve microcirculation [5]. Current literature lacks rigorous assessments of these effects, despite the technique’s global adoption [6]. This gap is critical because subclinical microvascular changes detectable through optical coherence tomography angiography (OCTA) may precede overt vision loss, offering a window for preventive intervention [7]. The low number of reported visual complications in UBE surgery should not be interpreted as definitive evidence of ocular safety, as subclinical changes may go undetected when targeted imaging modalities are not employed. In this context, OCTA may provide a noninvasive and sensitive means of assessing retinal and optic nerve microcirculation, thereby allowing hypotheses related to ocular safety to be explored even in the absence of overt clinical findings.

This study seeks to characterize the impact of UBE’s hydrostatic dynamics on retinal and optic nerve integrity while proposing evidence-based safeguards. By correlating intraoperative parameters with OCTA-derived microvascular metrics, we aim to delineate pressure thresholds that balance surgical efficacy with ocular safety. The findings are intended to refine clinical protocols, reducing POVL risks without compromising the technical advantages of UBE.

## 2. Materials and Methods

This study was a retrospective cohort study. Ethical approval was obtained from the local ethics committee. The data were retrieved from the hospital’s electronic archives and the clinical archives, where detailed manual recordings of patient measurements were maintained. The study was conducted in the departments of orthopedics and ophthalmology of a university hospital. At the initial period when UBE surgery was introduced in our institution, all patients routinely underwent ophthalmologic examinations including OCTA as part of a precautionary clinical practice to monitor for potential ocular complications. This was a local clinical approach initiated for patient safety. However, this routine ophthalmologic examination practice was abandoned after no ocular complications were observed in these patients, and it is no longer implemented in our current clinical protocol. The examinations and images were documented in the medical records, and the present study retrospectively analyzed these existing data. The retrospective study included patients treated between December 2022 and April 2024. A total of 55 patients who underwent surgery due to lumbar stenosis and had ophthalmologic examinations during both preoperative and postoperative follow-up were included in the study. Of these, 8 patients with diabetes mellitus, 10 with hypertension, 4 with a history of ocular surgery, and 1 with a history of ocular vascular occlusion were excluded. The data of the remaining 32 patients were analyzed. The mean age of the patients was 67.6 ± 7.3 years. Of the patients, 22 were female and 10 were male. Surgical decompression was performed at a single level in 18 patients, at two levels in 8 patients, and at three levels in 6 patients. The mean duration of surgery was 81.7 ± 32.3 min.

### 2.1. Surgical Technique

All patients were positioned prone on silicone padding with their eyes completely free of external pressure. The head was placed in slight extension (approximately 5–10 degrees), and the operating table was adjusted to a mild reverse Trendelenburg position to facilitate venous drainage while maintaining optimal surgical exposure. Intraoperative blood pressure was closely monitored and maintained within a systolic range of 90–100 mmHg and a diastolic range of 50–60 mmHg, with a mean arterial pressure of approximately 70 mmHg throughout the procedure. UBE procedure was performed through two distinct paramedian portals (viewing and working) created via 1 cm incisions under fluoroscopic guidance. A 0° arthroscope was introduced through the viewing portal while surgical instruments were manipulated through the working portal, with continuous irrigation. In all cases, irrigation was performed using isotonic saline solution, which was maintained at a constant height of 60 cm above the surgical field, thereby providing a uniform and gravity-controlled irrigation pressure across all patients. This irrigation height corresponded to an estimated hydrostatic pressure of approximately 30–50 mmHg, and no pressure-regulated pump systems were used. Particular attention was given to maintaining an appropriate 2–3 cm interportal distance to prevent instrument crowding while preserving adequate working space. Meticulous hemostasis was achieved using bipolar coagulation. Due to the minimally invasive nature of the procedure, no clinically significant intraoperative blood loss was observed, and no patients required blood transfusion. Neural structures were carefully manipulated with minimal traction to reduce iatrogenic injury risk while ensuring sustained visualization quality, in accordance with established UBE protocols [3,6]. These technical considerations collectively served to prevent visual complications and maintain surgical precision throughout the procedure.

### 2.2. Ophthalmologic Examinations

**Figure 1:**
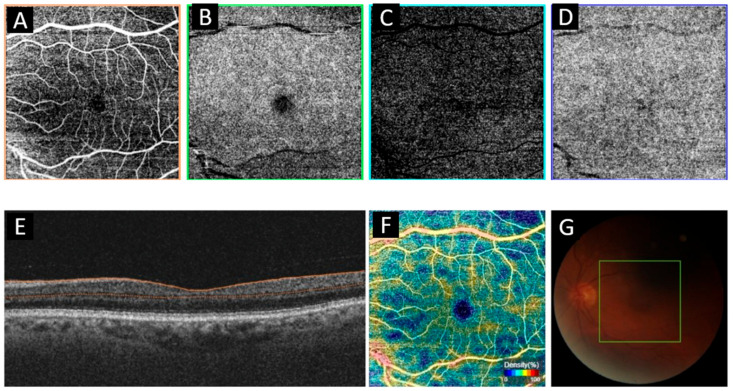
Optical Coherence Tomography Angiography (OCTA) is used to identify blood vessels in the retinal layers. The superficial layer (**A**) shows a well-defined retinal vasculature, while the middle layer (**B**) and deep layer (**C**) appear denser and more granular. The choriocapillaris layer, devoid of large vessels, appears hyperreflective (**D**). A cross-sectional OCT B-scan image (**E**) shows each retinal layer in detail. The vascular density is automatically calculated by the device (**F**). The fundus photograph of the right eye (**G**) shows the optic disc and macula, with a 6 × 6 mm green square and horizontal/vertical lines indicating the scan area for OCTA imaging.

The participants’ ophthalmologic examinations were performed by a senior ophthalmologist (AB). All measurements were performed before surgery, at 1 week and 4 week after surgery. Routine eye examinations such as refraction measurements and visual acuity, biomicroscopic examination, intraocular pressure and fundus examination were provided by the same ophthalmologist. Both eyes of the participants were examined, but only the data of the right eye were included. After a 15 min rest period for the patients, we used the swept-source deep-range imaging (DRI) optical coherence tomography (OCT) Triton device to capture OCTA images (Figure 1). All subjects acquired OCTA using a swept-source OCT (DRI OCT Triton; Topcon Inc., Tokyo, Japan) with a wavelength of 1050 nm, an acquisition rate of 100,000 A-scans per second, and axial and transverse resolutions of 7 and 20 mm in tissue, respectively. To obtain OCTA images, we performed a 6 × 6 mm volumetric scan centered on the fovea. A skilled technician measured all parameters with the Triton device. OCTA images were screened for (1) quality score < 40, (2) motion artifacts, (3) incorrect segmentation of tissue layers or sheets, (4) blurry images (inability to distinguish fine capillary networks from background signal), (5) signal loss, (6) poor centration, and inappropriate images were excluded from the study. All images were meticulously quality-controlled to ensure segmentation accuracy in the study, and images with segmentation errors were corrected manually or removed from the analysis when necessary. Segmentation was performed with the device’s IMAGEnet6 software (v1.14.8538), and quality control was performed by an operator (AB) experienced with the device and segmentation software. The best image was selected. The built-in software (IMAGEnet6, v1.14.8538) automated and segmented the superficial capillary plexus (SCP) and deep capillary plexus (DCP) sheets; The SCP was drawn from 2.6 mm below the internal limiting membrane to 15.6 mm below the junction between the inner plexiform and inner nuclear layers; the DCP was drawn from 15.6 mm below the inner plexiform and 70.2 mm below the inner nuclear layers. The ophthalmologist also measured the vascular density (VD), retinal nerve fiber layer (RNFL), foveal avascular zone (FAZ), central macular thickness (CMT), and subfoveal choroidal thickness (SFCT) with the OCTA device [8].

### 2.3. Statistics

Statistical analysis was performed using the SPSS 22 software (IBM, Armonk, NY, USA). Repeated measures analysis of variance (ANOVA) was conducted using the General Linear Model to evaluate changes in at three time points: preoperative, postoperative week 1, and postoperative week 4. The within-subject factor was defined as “time” with three levels. Mauchly’s test of sphericity was used to assess the assumption of sphericity. If the sphericity assumption was violated (*p* < 0.05), Greenhouse-Geisser values were used; otherwise, the “Sphericity Assumed” results were interpreted. When the overall time effect was statistically significant, post hoc pairwise comparisons between time points were performed using the Bonferroni method. A *p*-value of less than 0.05 was considered statistically significant.

## 3. Results

In the postoperative period, none of the patients experienced any vision-related problems. A statistically significant difference across the three time points (preoperative, postoperative week 1, and postoperative week 4) was found in FAZs (*p* = 0.043), VDd superior (*p* = 0.018), VDd temporal (*p* = 0.032), and RNFLts (*p* = 0.032). In contrast, the other parameters did not show statistically significant changes between the time points (*p* > 0.05) (Table 1) (Supplementary Table S1). In the pairwise comparisons between preoperative, postoperative week 1, and postoperative week 4, no statistically significant differences were found for FAZs, VDd temporal, and RNFLts (*p* > 0.05). However, VDd superior measurements were significantly lower at postoperative week 4 compared to the preoperative period (*p* = 0.050) (Table 2).

## 4. Discussion

UBE spinal surgery has emerged as an effective minimally invasive alternative to conventional open techniques for lumbar disc herniation and spinal stenosis [1,6]. While the benefits of UBE regarding tissue preservation and postoperative recovery are well-documented [3], concerns persist about potential ocular complications related to the hydrostatic pressure required for irrigation during the procedure [2]. In this study, UBE spinal surgery was not associated with clinically evident visual loss or sustained OCTA-detected microvascular alterations during short-term follow-up.

The key finding of our study was the statistically significant but clinically insignificant reduction in superior deep capillary plexus vascular density (VDd superior) at four weeks postoperatively. While this observation might theoretically suggest microvascular compromise, several factors indicate it lacks clinical relevance. First, no patient in our cohort experienced any visual symptoms or detectable functional impairment. Second, the changes were limited to specific OCTA parameters without corresponding alterations in visual acuity, intraocular pressure, or retinal nerve fiber layer thickness. Third, the magnitude of change was modest and within normal physiological variation [7]. Taken together, these observations suggest that UBE surgery was not associated with overt or clinically relevant impairment of ocular perfusion parameters. The absence of clinically relevant microvascular impairment on OCTA in the present study should be interpreted as part of a hypothesis-testing approach rather than as definitive exclusion of risk. When evaluating rare but potentially serious complications such as POVL, even the absence of detectable short-term changes may still provide useful information regarding surgical safety. In the context of relatively novel surgical techniques such as UBE, the inability to demonstrate measurable microvascular damage using objective imaging modalities may offer preliminary safety-related insight for the existing literature. However, these findings should be interpreted with caution, as they are based on a limited patient cohort, and surgeons should remain mindful of potential risks.

Given the well-documented association between open spine surgery and POVL [5,9], concerns regarding ophthalmological safety remain relevant in spinal procedures. Ischemic optic neuropathy has been identified as the most common mechanism underlying POVL in this setting [10]. In the present study, which evaluated subtle perioperative ocular changes in a limited cohort, no cases of clinically apparent vision loss were observed. Consistent with this, no clinical vision loss has been reported in the existing UBE literature to date [11,12]. While the current findings do not allow for direct comparisons or definitive risk reduction claims, they suggest that the controlled irrigation environment of UBE does not appear to introduce additional ophthalmological risk within the parameters assessed. The transient nature of the observed microvascular changes deserves particular emphasis. Overall, no consistent or clinically meaningful differences were detected in OCTA parameters across the postoperative follow-up period. Only VDd superior showed a persistent but minor reduction at four weeks. This pattern suggests a temporary physiological adjustment rather than permanent microvascular damage. Similar transient changes have been reported in other surgical procedures requiring prone positioning [13,14], indicating they may represent a systemic response to surgical stress rather than a UBE-specific effect.

From a technical perspective, several elements of our surgical protocol likely played a role in achieving these favorable outcomes. Thoughtful portal placement to reduce instrument crowding, together with meticulous hemostasis, constitutes important protective measures [15,16]. Our observations indicate that strict adherence to these technical details may be more influential in preventing ocular complications than the absolute level of irrigation pressure itself. This interpretation is consistent with studies demonstrating that well-regulated fluid dynamics in UBE can provide a stable operative field without generating hazardous pressure fluctuations [17,18]. In the present study, UBE spinal surgery was not associated with clinically evident vision loss or sustained OCTA-detected microvascular alterations during short-term follow-up. While these findings are reassuring, they should not be interpreted as definitive evidence of absolute ocular safety. Therefore, based on the scope of the present study, the findings may suggest that UBE performed under controlled technical and hemodynamic conditions is unlikely to be associated with clinically meaningful visual complications in low-risk patients; however, larger prospective studies are needed to confirm these observations. Nevertheless, a cautious approach remains appropriate. Patients with preexisting vascular conditions such as diabetes or hypertension, as well as those with ocular diseases including glaucoma, may have diminished microvascular reserve [19,20], which could increase their susceptibility to pressure-related effects. In this context, a preoperative ophthalmologic assessment and closer postoperative follow-up may be considered. Moreover, surgeons should remain attentive to maintaining appropriate irrigation pressure and limiting operative duration, particularly in complex cases that involve prolonged procedures [21,22].

This study has several limitations. First, ophthalmological examinations and OCTA measurements were performed as a precautionary approach to monitor potential ocular complications during the initial period when unilateral biportal endoscopic surgery was first implemented at our institution. As no ocular complications were detected in clinical practice during this period, this assessment was later removed from the routine protocol. Therefore, the study group consists of a limited patient population from the early phase of surgical implementation, which may introduce potential selection bias. The retrospective design of the study and the relatively small sample size are additional factors that limit the generalizability and statistical power of the results. Moreover, the absence of a control group makes it difficult to definitively determine whether the observed OCTA changes are specific to this surgical procedure. Due to the follow-up period being limited to four weeks, potential late-onset microvascular changes could not be evaluated. Finally, the lack of clearly defined clinically meaningful thresholds for OCTA parameters in the literature restricts the interpretation of the clinical relevance of some findings that were statistically significant. Future studies with larger patient cohorts, prospective designs, inclusion of control groups, and longer follow-up periods would help strengthen the evidence in this field.

## 5. Conclusions

In this retrospective single-center series, UBE spinal surgery was not associated with clinically evident vision loss or sustained OCTA-detected retinal microvascular changes during short-term follow-up. Although minor, transient OCTA variations were observed, no persistent microstructural or functional ocular impairment was detected. These findings should be interpreted as preliminary and hypothesis-generating, underscoring the need for prospective, controlled studies with longer follow-up to more definitively assess ocular safety.

## Figures and Tables

**Table 1:** Comparison of Measured Parameters Across Preoperative and Postoperative Time Points.

	Preop	Postop 1 w	Postop 4 w	*p* Value
VA	20/20	20/20	20/20	1
IOP	15.84 ± 2.58	16.22 ± 2.32	15.84 ± 2.71	0.803
FAZs	242.43 ± 67.70	242.86 ± 63.20	264.31 ± 62.47	0.043 *
VDs central	20.88 ± 3.84	20.85 ± 3.79	20.79 ± 4.31	0.926
VDs superior	50.10 ± 2.99	49.96 ± 2.93	50.57 ± 3.44	0.268
VDs temporal	46.11 ± 2.83	46.18 ± 2.92	45.58 ± 3.38	0.406
VDs inferior	49.12 ± 3.89	49.33 ± 3.86	48.80 ± 2.67	0.465
VDs nasal	44.88 ± 3.61	44.85 ± 3.59	45.64 ± 4.31	0.228
FAZd	227.65 ± 60.56	227.65 ± 60.56	239.03 ± 60.72	0.224
VDd central	20.85 ± 4.43	20.88 ± 4.46	21.24 ± 5.04	0.638
VDd superior	52.28 ± 2.66	52.22 ± 2.63	51.17 ± 2.63	0.018 *
VDd temporal	48.29 ± 4.25	48.28 ± 4.19	46.10 ± 5.12	0.032 *
VDd inferior	52.40 ± 4.28	52.46 ± 4.32	50.92 ± 4.95	0.103
VDd nasal	49.07 ± 4.21	49.05 ± 4.20	47.25 ± 5.13	0.069
RNFL mean	102.53 ± 11.75	102.53 ± 11.84	100.31 ± 10.19	0.299
RNFL nasal superior	132.09 ± 23.15	131.22 ± 21.66	134.50 ± 23.24	0.538
RNFL temporal superior	105.91 ± 17.93	106.50 ± 17.46	100.34 ± 12.20	0.032 *
RNFL temporal	80.41 ± 12.49	80.59 ± 11.79	80.63 ± 12.66	0.943
RNFL temporal inferior	98.81 ±17.74	98.81 ± 17.87	97.28 ± 11.94	0.535
RNFL nasal inferior	148.63 ± 21.73	148.09 ± 22.00	144.34 ± 20.69	0.355
RNFL nasal	88.41 ± 17.63	88.41 ± 18.00	89.03 ± 17.90	0.787
CMT	268.38 ± 30.19	263.19 ± 23.10	260.75 ± 22.76	0.143
SCFT	323.94 ± 60.16	324.16 ± 59.89	320.28 ± 50.27	0.711

Data presented as mean + standard deviation. VA: Visual acuity; IOP: Intraocular Pressure; FAZ s/d: Foveal Avascular Zone (superficial/deep); VD s/d: Vascular Density (superficial/deep); RNFL: Retinal Nerve Fiber Layer; CMT: Central Macular Thickness; SCFT: Subfoveal Choroidal Thickness. Values marked with an asterisk (*) and shown in bold indicate *p* < 0.05.

**Table 2:** Pairwise Comparison of Measurements Over Time.

**FAZs**		***p* Value**
Preop	Postop 1 w	0.508
	Postop 4 w	0.124
Postop 1 w	Preop	0.508
	Postop 4 w	0.136
Postop 4 w	Preop	0.124
	Postop 1 w	0.136
**VDd Superior**		***p* Value**
Preop	Postop 1 w	0.296
	Postop 4 w	0.050 *
Postop 1 w	Preop	0.296
	Postop 4 w	0.060
Postop 4 w	Preop	0.050 *
	Postop 1 w	0.060
**VDd Temporal**		***p* Value**
Preop	Postop 1 w	1.000
	Postop 4 w	0.095
Postop 1 w	Preop	1.000
	Postop 4 w	0.097
Postop 4 w	Preop	0.095
	Postop 1 w	0.097
**RNFLts**		***p* Value**
Preop	Postop 1 w	0.162
	Postop 4 w	0.127
Postop 1 w	Preop	0.162
	Postop 4 w	0.076
Postop 4 w	Preop	0.127
	Postop 1 w	0.076

Values marked with an asterisk (*) and shown in bold indicate *p* < 0.05.

## Data Availability

The raw data supporting the conclusions of this article will be made available by the authors on request.

## Supplementary Materials

The following supporting information can be downloaded at: https://www.mdpi.com/article/10.3390/jcm15051774/s1. Table S1. Comparison of Measured Parameters Across Preoperative and Postoperative Time Points.

## Abbreviations

The following abbreviations are used in this manuscript:

CMT	Central Macular Thickness
DCP	Deep Capillary Plexus
DRI	Deep-Range Imaging
FAZ	Foveal Avascular Zone
ION	Ischemic Optic Neuropathy
OCT	Optical Coherence Tomography
OCTA	Optical Coherence Tomography Angiography
OVL	Perioperative Vision Loss
RNFL	Retinal Nerve Fiber Layer
SCP	Superficial Capillary Plexus
SFCT	Subfoveal Choroidal Thickness
UBE	Unilateral Biportal Endoscopic (Surgery)
VD	Vascular Density
VDd	Deep Capillary Plexus Vascular Density
