# Supplementary material for: Ocular Safety of Unilateral Biportal Endoscopic Spinal Surgery: An Optical Coherence Tomography Angiography-Based Analysis
Source: J Clin Med. 2026 Feb 26;15(5):1774. doi: 10.3390/jcm15051774 (PMC12985841; doi:10.3390/jcm15051774)
Supplement: Supplementary file 1 [file jcm-15-01774-s001.zip › jcm-4132752-supplementary.pdf]

Table S1. Comparison of Measured Parameters Across Preoperative and Postoperative Time Points

	Preop	Postop 1w	Postop 4w	p
VA	20/20	20/20	20/20	1
IOP	15.84 ± 2.58 (95% CI: 14.91-16.77)	16.22 ± 2.32 (95% CI:15.38-17.06)	15.84 ± 2.71 (95% CI: 14.87-16.82)	0.803
FAZ s	242.43 ± 67.70 (95% CI: 219.82-265.03)	242.86 ± 63.20 (95% CI:220.07-265.65)	264.31 ± 62.47 (95% CI: 241.78-286.82)	**0.043 ***
VDs central	20.88 ± 3.84 (95% CI: 19.49-22.26)	20.85 ± 3.79 (95% CI: 19.48-22.21)	20.79 ± 4.31 (95% CI:19.23-22.34)	0.926
VDs superior	50.10 ± 2.99 (95% CI: 49.02-51.17)	49.96 ± 2.93 (95% CI: 48.90-51.01)	50.57 ± 3.44 (95% CI: 49.33-51.81)	0.268
VDs temporal	46.11 ± 2.83 (95% CI: 45.08-47.12)	46.18 ± 2.92 (95% CI: 45.13-47.23)	45.58 ± 3.38 (95% CI: 44.36-46.79)	0.406
VDs inferior	49.12 ± 3.89 (95% CI: 47.72-52.52)	49.33 ± 3.86 (95% CI: 47.94-50.72)	48.80 ± 2.67 (95% CI: 47.83-49.76)	0.465
VDs nasal	44.88 ± 3.61 (95% CI: 43.58-46.18)	44.85 ± 3.59 (95% CI:43.55-46.14)	45.64 ± 4.31 (95% CI: 44.08-47.19)	0.228
FAZ d	227.65 ± 60.56 (95% CI: 205.81-249.48)	227.65 ± 60.56 (95% CI:205.81-249.48)	239.03 ± 60.72 (95% CI: 217.13-260.92)	0.224
VDd central	20.85 ± 4.43 (95% CI: 19.25-22.45)	20.88 ± 4.46 (95% CI: 19.27-22.48)	21.24 ± 5.04 (95% CI: 19.42-23.05)	0.638
VDd superior	52.28 ± 2.66 (95% CI: 51.31-53.23)	52.22 ± 2.63 (95% CI: 51.27-53.17)	51.17 ± 2.63 (95% CI: 50.22-52.12)	**0.018***
VDd temporal	48.29 ± 4.25 (95% CI: 46.75-49.82)	48.28 ± 4.19 (95% CI: 46.77-49.79)	46.10 ± 5.12 (95% CI: 44.25-47.94)	**0.032***
VDd inferior	52.40 ± 4.28 (95% CI: 50.85-53.93)	52.46 ± 4.32 (95% CI: 50.90-54.01)	50.92 ± 4.95 (95% CI:19.13-52.70)	0.103
VDd nasal	49.07 ± 4.21 (95% CI: 47.55-50.58)	49.05 ± 4.20 (95% CI: 47.53-50.56)	47.25 ± 5.13 (95% CI: 45.39-49.09)	0.069
RNFL mean	102.53 ± 11.75 (95% CI: 98.29-106.77)	102.53 ± 11.84 (95% CI: 98.26-106.80)	100.31 ± 10.19 (95% CI: 96.64-103.99)	0.299
RNFL nasal superior	132.09 ± 23.15 (95% CI: 123.75-140.44)	131.22 ± 21.66 (95% CI: 123.41-139.03)	134.50 ± 23.24 (95% CI:126.12-142.88)	0.538
RNFL temporal superior	105.91 ± 17.93 (95% CI: 99.44-.112.37)	106.50 ± 17.46 (95% CI: 100.21-112.79)	100.34 ± 12.20 (95% CI: 95.94-104.74)	**0.032***

RNFL temporal	80.41 ± 12.49 (95% CI: 75.+0-84.91)	80.59 ± 11.79 (95% CI:76.34-84.84)	80.63 ± 12.66 (95% CI: 76.06-85.19)	0.943
RNFL temporal inferior	98.81 ±17.74 (95% CI:92.41-105.21)	98.81 ± 17.87 (95% CI: 92.37-105.26)	97.28 ± 11.94 (95% CI: 92.98-101.59)	0.535
RNFL nasal inferior	148.63 ± 21.73 (95% CI:140.79-156.46)	148.09 ± 22.00 (95% CI: 140.16-156.03)	144.34 ± 20.69 (95% CI: 136.89-151.80)	0.355
RNFL nasal	88.41 ± 17.63 (95% CI: 82.05-94.76)	88.41 ± 18.00 (95% CI: 81.92-94.90)	89.03 ± 17.90 (95% CI: 82.58-95.48)	0.787
CMT	268.38 ± 30.19 (95% CI: 257.49-279.26	263.19 ± 23.10 (95% CI: 254.86-271.52)	260.75 ± 22.76 (95% CI: 252.55-268.95)	0.143
SCFT	323.94 ± 60.16 (95% CI: 302.25-345.63)	324.16 ± 59.89 (95% CI: 302.57-345.75)	320.28 ± 50.27 (95% CI: 302.16-338.41)	0.711

Data presented as mean ± standard deviation (95% CI)

VA: Visual acuity
IOP: Intraocular Pressure
FAZ s/d: Foveal Avascular Zone (superficial / deep)
VD s/d: Vascular Density (superficial / deep)
RNFL: Retinal Nerve Fiber Layer
CMT: Central Macular Thickness
SFCT: Subfoveal Choroidal Thickness
